# Antibiotic Resistance in *Bifidobacterium animalis* subsp. *lactis* and *Bifidobacterium longum*: Definition of Sensitivity/Resistance Profiles at the Species Level

**DOI:** 10.3390/microorganisms13071647

**Published:** 2025-07-11

**Authors:** Mario Terlizzi, Barbara Speranza, Milena Sinigaglia, Maria Rosaria Corbo, Antonio Bevilacqua

**Affiliations:** Department of Agriculture, Food, Natural Resources and Engineering, University of Foggia, Via Napoli 25, 71122 Foggia, Italy; mario.terlizzi@unifg.it (M.T.); barbara.speranza@unifg.it (B.S.); milena.sinigaglia@unifg.it (M.S.); mariarosaria.corbo@unifg.it (M.R.C.)

**Keywords:** antibiotic resistance, *Bifidobacterium* spp., MIC values, probiotics, susceptibility

## Abstract

Antimicrobial resistance is a threat to probiotic microorganisms due to their potential role in harboring and transmitting resistance genes. This study focuses on two *Bifidobacterium* species (*B. animalis* subsp. *lactis* and *B. longum*) by analyzing 657 Minimal Inhibitory Concentration (MIC) values extracted from research articles indexed in Scopus, PubMed, and Web of Science, published since 2014, and considering 17 different antibiotics. MIC values were used for descriptive statistical analysis (boxplots and violin plots) to evaluate both inter- and intraspecies distributions. The results showed an overall increase in MIC values compared to historical data, with *B. longum* exhibiting high resistance to tetracyclines and streptomycin—approximately 25% to 50% of the strains had MIC values > EFSA cut-offs. The violin plots revealed the presence of resistant subpopulations, particularly within *B. longum*. These findings support the relevance of longitudinal MIC analysis as a tool for detecting early shifts in antimicrobial susceptibility and highlight the importance of data-driven approaches for microbiological risk assessment in probiotic applications.

## 1. Introduction

Antimicrobial resistance (AMR), generally defined as the resistance or ability/possibility of a microorganism to counteract the effects of antimicrobial agents, is a global threat. Its implications can be severe, as timely administration of effective antimicrobials remains the most critical intervention for reducing the risk of adverse outcomes in serious infections [1]. AMR emerges naturally from mutations in bacterial genes; indeed, bacteria can develop multiple resistance mechanisms, rendering them resistant to various antimicrobial compounds. This significantly limits available treatment options for infectious diseases [1].

The World Health Organization has listed AMR among the ten greatest threats to global public health [2], as the inability to treat infections with antibiotics raises serious concerns for the future of healthcare systems [3]. AMR is also relevant in the context of probiotic microorganisms, which may act as reservoirs or vectors for resistance determinants, especially when such traits are transferable to pathogenic bacteria [4]. Among probiotics, *Bifidobacterium* spp. is one of the most studied and widely used genera, with a long-standing history of safe use in humans [5]. However, even these beneficial microorganisms are not exempt from the risks associated with AMR [6].

One study investigated the antibiotic susceptibility of various *Bifidobacterium* strains, including *B. bifidum*, *B. longum* subsp. *longum*, and *B. animalis* subsp. *lactis*. All the strains were sensitive to penicillins, whereas the majority (70%) showed resistance to fusidic acid. Acquired resistance was observed in 14% of the strains, specifically to tetracyclines and minocycline [7].

Another study analyzed the antibiotic susceptibility of fifty *Bifidobacterium* strains, representing eight species, against a panel of thirty antimicrobial agents. Minimimal Inhibitory Concentrations (MICs) for nine compounds were evaluated using the reference agar dilution method [8], suggesting the possibility of horizontal gene transfer in bifidobacteria, as seen in other intestinal commensals [9].

The accurate determination of MIC and Minimum Bactericidal Concentration (MBC) values is essential for assessing antibiotic efficacy against bifidobacteria and for tracking resistance development. These data are critical in guiding the selection of appropriate antimicrobial therapies [10]. However, no comprehensive study currently encompasses all *Bifidobacterium* strains. Most existing research focuses on individual strains, making it difficult to define consistent resistance or susceptibility profiles across species and subspecies.

Therefore, the present research aimed to examine antibiotic resistance in food-associated microorganisms, focusing specifically on the genus *Bifidobacterium*, which includes numerous probiotic strains. The goal was to quantitatively assess their resistance to selected commonly used antibiotics based on MIC values and to evaluate the implications for consumer health.

The specific objectives were as follows: (i) to investigate antibiotic resistance within the genus *Bifidobacterium*, focusing on *B. animalis* subsp. *lactis* and *B. longum*, by analyzing over 600 MIC values; (ii) to evaluate resistance and susceptibility profiles using the European Food Safety Authority (EFSA) reference cut-off values; and (iii) to compare the findings with current antibiotic usage to assess potential risk.

## 2. Materials and Methods

### 2.1. Data Selection and Acquisition

The first step of this research involved a literature search in PubMed, Scopus, and Web of Science using the following keywords: *Bifidobacterium*, antibiotic resistance, and MIC. Only articles published from 2014 onward were considered. The following inclusion criteria were applied: (i) MIC data had to be expressed in μg/mL or convertible units of measurement. (ii) Because of the high number of species, the analysis focused exclusively on *B. animalis* subsp. *lactis* and *B. longum*. (iii) MIC evaluations had to be performed using E-test, micro-dilution, or similar standardized protocols. (iv) Only antibiotics with MIC values available for at least 4–5 strains were included in the second step (statistical analyses described in Section 2.2). Data extraction was performed manually when tables were available in the manuscripts. In cases where MIC values were reported in a graphical form, the WebPlotDigitizer online tool (https://automeris.io/, accessed on 4 July 2025) was used. As a result, MIC data for 17 different antibiotics across the two species were collected, yielding a total of 657 records after the removal of duplicates.

### 2.2. Statistics

Data retrieved from the literature were organized in a spreadsheet, reporting the MIC values found for each antibiotic and each species. These were then used to calculate the mean, standard deviation, and 1st and 3rd quartiles. Box–whisker graphs and violin plots were generated to visualize distribution patterns and identify interspecies differences (*t*-test, *p* < 0.05). Statistical analyses were performed using Statistica for Windows version 12.5 (Statsoft, Tulsa, OK, USA) and PAST version 5.2.2 (https://www.nhm.uio.no/english/research/resources/past/ accessed on 4 June 2025).

## 3. Results and Discussion

The rate at which antibiotic-resistant bacterial strains emerge often exceeds the pace of development of new active compounds, making it a serious problem for healthcare systems [11]. The clinical implications are significant and include increased mortality, prolonged illness duration, the onset of complications, and the risk of epidemics [12]. Additionally, antibiotic resistance results in elevated economic costs due to hospitalization and the need for next-generation drugs, which are typically more expensive [12].

The phenomenon of antibiotic resistance is steadily increasing and represents a growing threat to public health. This trend is supported by data from the Culture Collection of Switzerland (CCOS), a Swiss center specializing in the collection and analysis of microbial strains. The CCOS has standardized antibiotic resistance testing for bifidobacteria and lactic bacteria, in accordance with ISO 10932. However, a comprehensive overview of the different resistance profiles of bifidobacterial strains is still lacking. For this reason, the historical data were used to build sensitivity profiles for the two *Bifidobacterium* species.

The following figures present the data analysis for *Bifidobacterium animalis* subsp. *lactis* and *B. longum*. The data were interpreted based on EFSA cut-off values, which indicate whether a strain is sensitive or resistant to an antibiotic. This evaluation is the first step for a safety assessment of a food-grade microorganism, mainly for its antibiotic resistance profile [13].

**Figure 1:**
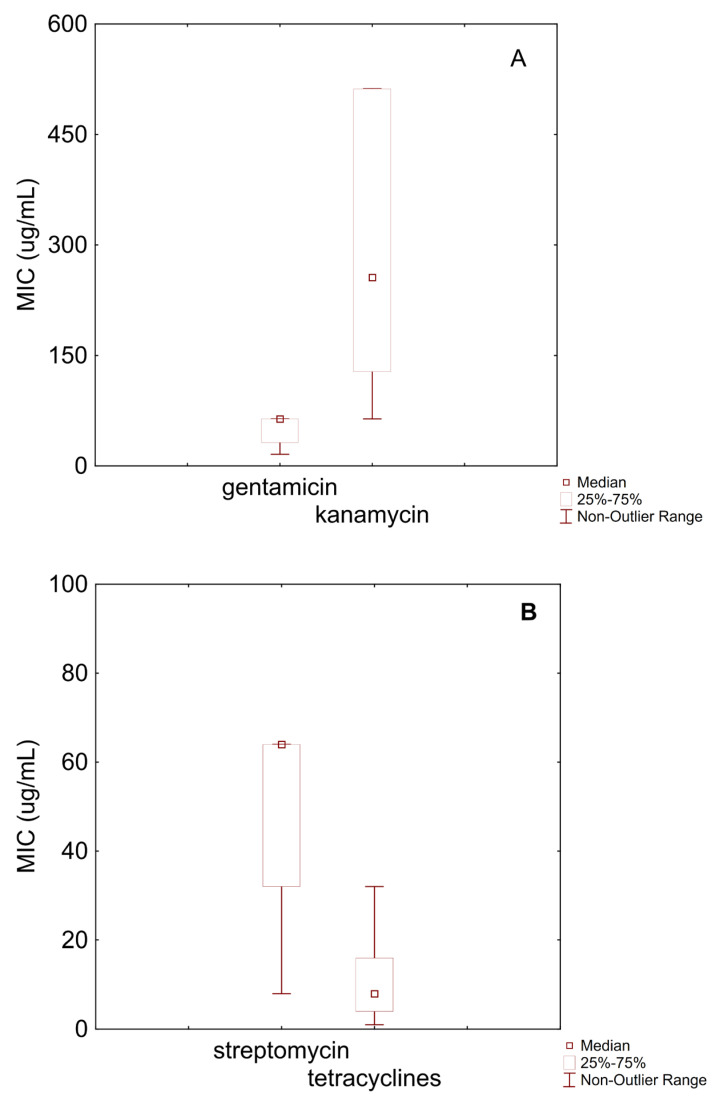

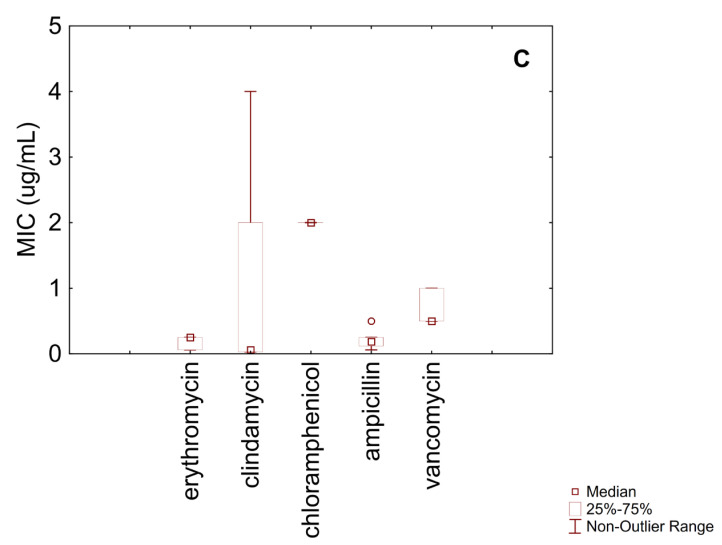
Box–whisker diagram for MIC values of antibiotics against *B. animalis* subsp. *lactis*. (**A**) MIC for gentamicin and kanamycin; (**B**) streptomycin, and tetracyclines; (**C**) erythromycin, clindamycin, chloramphenicol, ampicillin, and vabcomycin.

Figure 1A shows boxplots for gentamicin and kanamycin against *B. animalis* subsp. *lactis*. Gentamicin exhibited a median MIC of 64 µg/mL, with an interquartile range (IQR) between 32 µg/mL and 64 µg/mL and a minimum value of 16 µg/mL. Based on the EFSA microbiological cut-off (64 µg/mL), approximately 75% of the examined strains were classified as susceptible, while around 25% may be considered resistant. The MIC distribution for kanamycin appeared more heterogeneous, with a median of 256 µg/mL, an IQR between 128 µg/mL and 512 µg/mL, and a range from 64 µg/mL to 512 µg/mL. Susceptibility could not be determined for kanamycin, as the EFSA has not defined a cut-off value.

Figure 1B reports MIC data for streptomycin and tetracyclines. Streptomycin had a median MIC of 64 µg/mL, with values ranging from 8 µg/mL to 128 µg/mL. According to the EFSA cut-off (128 µg/mL), these results indicate a general susceptibility of *B. animalis* subsp. *lactis* to this antibiotic. Tetracyclines displayed a median MIC of 8 µg/mL, with an IQR from 4 µg/mL to 16 µg/mL. Based on the EFSA cut-off (8 µg/mL), approximately 50% of the strains could be considered susceptible.

Figure 1C shows MIC distributions for the remaining antibiotics tested against *B. animalis* subsp. *lactis*. Erythromycin had a relatively homogeneous distribution, with an IQR between 0.06 µg/mL and 0.25 µg/mL and a median MIC of 0.25 µg/mL. These values indicate general susceptibility, considering the EFSA cut-off (1 µg/mL). Clindamycin exhibited a median MIC of 0.06 µg/mL, with minimum and maximum values of 0.03 µg/mL and 2 µg/mL, respectively. Based on the EFSA cut-off (1 µg/mL), most strains were likely susceptible, although a small proportion may be resistant.

All MIC values for chloramphenicol were 2 µg/mL, indicating a highly homogeneous distribution and suggesting full susceptibility, as the EFSA cut-off is 4 µg/mL. Ampicillin showed a median MIC of 0.19 µg/mL and an IQR between 0.12 µg/mL and 0.31 µg/mL. Compared to the EFSA cut-off (2 µg/mL), all the tested strains were classified as susceptible.

Vancomycin exhibited an IQR between 0.5 µg/mL and 1 µg/mL, with a median MIC of 0.5 µg/mL. These values suggest overall susceptibility according to the EFSA cut-off (2 µg/mL).

**Figure 2:**
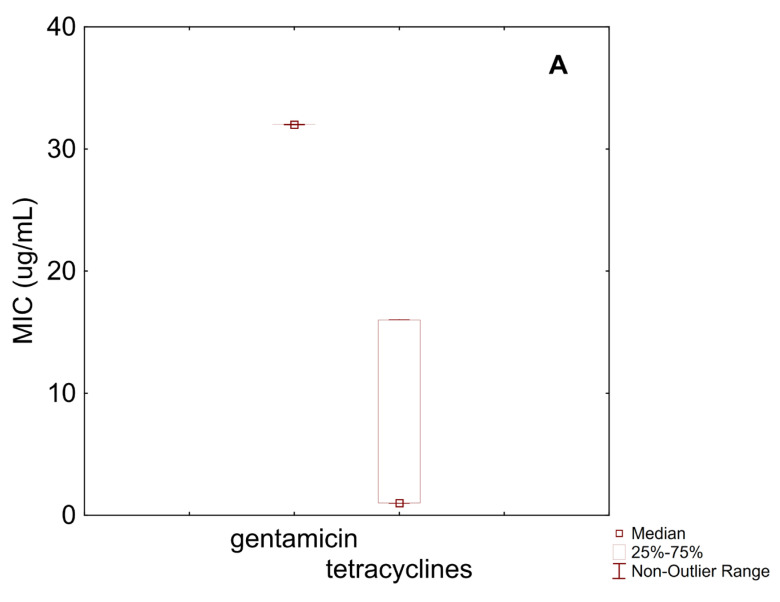

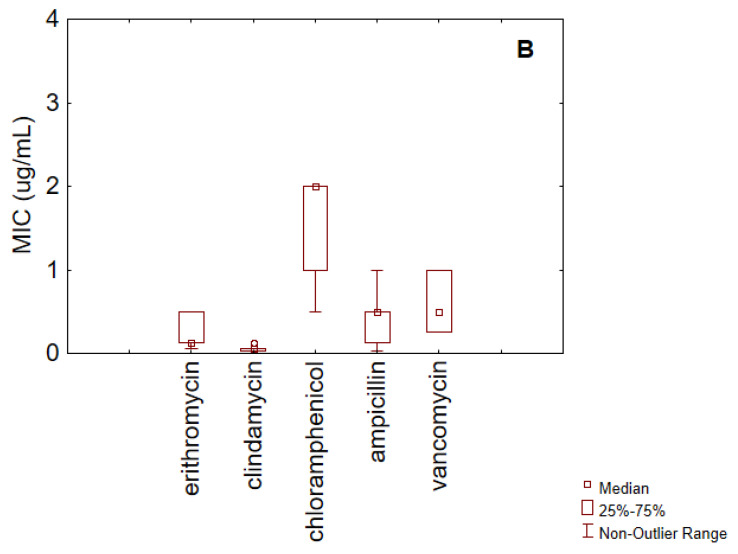
Box–whisker diagram for MIC values of antibiotics against *B. longum*. (**A**) gentamicin, and tetracyclines; (**B**) erythromycin, clindamycin, chloramphenicol, ampicillin, and vancomycin.

Figure 2A presents MIC data for gentamicin and tetracycline against *B. longum*. The MIC distribution for gentamicin was homogeneous, with 100% of the isolates exhibiting an MIC of 32 µg/mL, which is below the EFSA cut-off (64 µg/mL), indicating general susceptibility.

Tetracyclines showed a median MIC of 1 µg/mL and an IQR between 1 µg/mL and 16 µg/mL. Given the cut-off value (8 µg/mL), these results suggest that at least 25% of the strains may be considered resistant.

For streptomycin, the IQR ranged from 64 µg/mL to 512 µg/mL, with a median value of 128 µg/mL, which corresponds to the EFSA cut-off. Therefore, it can be inferred that at least 50% of the strains are resistant to this antibiotic.

For the remaining antibiotics (Figure 2B), erythromycin exhibited a median MIC of 0.13 µg/mL and an IQR between 0.13 µg/mL and 30 µg/mL. In light of the EFSA cut-off (1 µg/mL), this distribution suggests the presence of resistant strains.

Clindamycin had a median MIC of 0.03 µg/mL, with minimum and maximum values of 0.03 µg/mL and 0.06 µg/mL, respectively, indicating general susceptibility (EFSA cut-off: 1 µg/mL).

The MIC distribution for chloramphenicol was relatively homogeneous, with an IQR between 1 µg/mL and 2 µg/mL. The values ranged from 0.5 µg/mL to 4 µg/mL, and the median was 2 µg/mL. These findings suggest overall susceptibility based on the EFSA cut-off (4 µg/mL).

Ampicillin showed a median MIC of 0.50 µg/mL, with an IQR between 0.13 µg/mL and 0.50 µg/mL. The minimum and maximum values were 0.03 µg/mL and 1 µg/mL, respectively—all below the EFSA cut-off of 2 µg/mL.

Finally, vancomycin had a median MIC of 0.50 µg/mL and an IQR ranging from 0.25 µg/mL to 1 µg/mL, again indicating general susceptibility (cut-off: 2 µg/mL).

**Figure 3:**
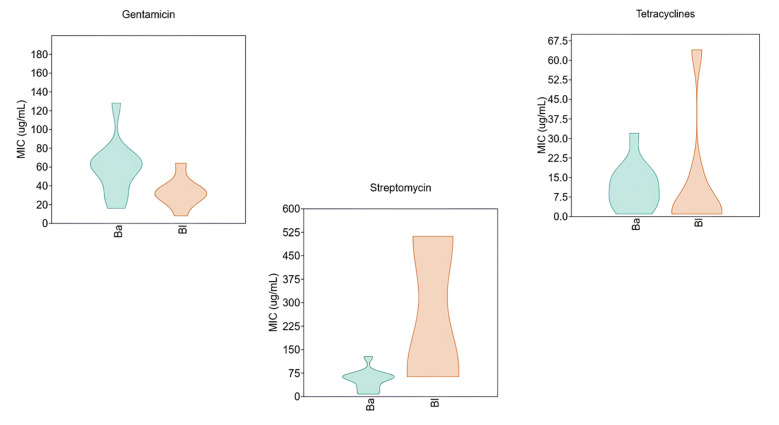
Violin diagram for MIC values of gentamicin, streptomycin, and tetracycline against *B. animalis* subsp. *lactis* (Ba) and *B. longum* (Bl).

Figure 3 focuses on the statistical distribution of MIC values for three selected antibiotics, comparing the two species and assessing potential subpopulations or differential susceptibility patterns within each species.

Regarding gentamicin, the two species exhibited markedly divergent susceptibility profiles. *B. animalis* subsp. *lactis* showed a broader MIC distribution, with most isolates presenting an MIC of 64 µg/mL. Notably, the violin plot revealed the presence of isolates with elevated resistance levels, reaching up to 128 µg/mL. In contrast, *B. longum* displayed a more homogeneous distribution, with MIC values predominantly centered around 32 µg/mL.

The differences were even more pronounced in the case of streptomycin, where the MIC distribution suggested the presence of two distinct subpopulations in *B. longum*: one with low resistance and another with high-level resistance. The latter exceeded the EFSA epidemiological cut-off. This bimodal distribution indicates a significant trend toward increased resistance to streptomycin in *B. longum*.

Finally, the violin plot for tetracyclines revealed a trend of rising resistance in *B. longum*. While most isolates exhibited MIC values below 7.5 µg/mL, the distribution displayed a noticeable “tailing effect,” with some values reaching up to 64 µg/mL.

A comparison of MIC values between the two *Bifidobacterium* species and the historical data from the Swiss database reveals an overall increase in antibiotic resistance over time. This trend even affects food-associated microorganisms, such as *Bifidobacterium*, with MIC values rising for most antibiotics analyzed [14]. While the dataset has some limitations, such as the geographical distribution of papers, i.e., some countries are not covered, strain origin (dairy products, humans, or other not-specified sources) or possible differences in the methodologies employed to gain the results, and the temporal range (only 10 years), it still provides a robust and representative sample of the resistance phenomenon in foodborne and food-grade microorganisms.

These findings raise significant concern. Although antibiotic-resistant probiotics may offer some benefits during therapy (survival, modulating gut microbiota, competitive exclusion of pathogens, reinforcing the intestinal barrier, preventing diarrhea, preventing antibiotic-associated diseases, like infections by *Clostridium difficile*, etc.) [15,16], these potential advantages must be carefully weighed against possible risks. The presence of resistance determinants on transferable elements (plasmids, transposons, or integrons) raises concerns about horizontal gene transfer to commensal or pathogenic bacteria, which may contribute to the spread of antimicrobial resistance [15,17,18,19]. Therefore, the selection of probiotic strains intended for use during antibiotic treatment should ensure not only functional efficacy but also the absence of transferable resistance determinants and should be based on a rigorous risk assessment [20]. The EFSA has clearly stated that the bacterial strains used in food and feed—such as starter cultures and probiotics—may only harbor antibiotic resistance traits if they are unequivocally shown to be of chromosomal origin and non-transmissible [21]. *Bifidobacterium* species have an intrinsic resistance to some antibiotics; for example, resistance to aminoglycosides is the result of the lack of active transport mechanisms required for antibiotic uptake, a typical feature of strict anaerobes [8]. Also, for β-lactam, intrinsic resistance was reported for some *Bifidobacterium* strains due to the reduced affinity of penicillin-binding proteins or the presence of low-activity β-lactamases [22].

Chromosomal resistance, typically caused by spontaneous mutations, is vertically transmitted and does not pose a risk of gene transfer. In contrast, extrachromosomal resistance, especially when associated with mobile genetic elements, represents a major biosafety concern due to its capacity for horizontal dissemination [23].

Acquired resistance genes have also been reported in some *Bifidobacterium* strains. For instance, the genes encoding resistance to tetracyclines (tet(W)) could be associated with plasmids or transposons, with evidence of a possible acquisition from other microorganisms [24], although, for some strains, it is a part of an ancient resistome [25]. The trend observed in *B. longum* suggests that tetracycline resistance may be increasing and deserves further monitoring.

Streptomycin resistance is likely due to chromosomal mutations [26], and the associated risk of gene transfer is minimal. However, the bimodal distribution suggests the necessity of a revision of the cut-off values in the International Guidelines.

Gentamicin resistance, by contrast, is plasmid-mediated [27,28], and the existence of strains with high MIC values poses a clear threat. Although violin plots were not created for all antibiotics, some compounds (e.g., kanamycin for *B. animalis*) showed wide boxplot distributions. This variability, regardless of the genetic origin of resistance, complicates the definition of clear susceptibility profiles at the species level.

It is critically important that probiotic strains intended for human consumption do not act as reservoirs of resistance genes [20]. This is especially relevant in vulnerable populations, such as infants, immuno-compromised individuals, or patients undergoing long-term antibiotic therapy, whose gut microbiota is more susceptible to perturbation. In such cases, the introduction of resistant probiotic strains could contribute to the dissemination of resistance traits in pathogenic bacteria [29].

Additionally, the use of antibiotic-susceptible probiotics offers a safety advantage. In the event of bacteremia or translocation of probiotic organisms across the intestinal barrier, these strains remain treatable with standard antibiotics. This is particularly important given the increasing reports of probiotic-associated infections in at-risk patient groups [30,31].

Another consideration involves comparing therapeutic concentrations of antibiotics to the MIC values observed in this study.

Gentamicin, typically administered parenterally at a daily dose of 350–490 mg for a 70 kg adult, showed MIC values of 64 µg/mL for *B. animalis* subsp. *lactis* and 32 µg/mL for *B. longum*, both below plasma concentrations achieved during therapy [32]. Similarly, streptomycin, administered at 1000 mg/day, corresponds to MIC values that remain within clinically relevant therapeutic windows [32]. These findings suggest that the strains analyzed in this study would not survive systemic antibiotic exposure and therefore do not present an increased risk of systemic persistence.

## 4. Conclusions

This study evaluated 657 MIC values for 17 antibiotics, extracted from the literature published since 2014, focusing on *B. animalis* subsp. *lactis* and *B. longum*. Statistical analysis revealed increasing MIC values in recent years; for instance, *B. longum* showed high MIC values for tetracyclines and streptomycin, with approximately 25% and 50% of the strains, respectively, exceeding EFSA epidemiological cut-offs. Violin plots also revealed potential subpopulations with elevated resistance.

In *B. animalis* subsp. *lactis*, resistance was less pronounced; 75% of the strains were susceptible to gentamicin, and nearly all were susceptible to erythromycin, clindamycin, and ampicillin. However, the high variability for kanamycin and streptomycin suggests strain-specific differences that warrant continued monitoring.

Although focused only on two species, the findings provide evidence of a shifting resistance profile in food-grade bifidobacteria. The approach proposed in this manuscript supports the implementation of data-driven microbiological risk assessment strategies and offers a scientific basis for updating regulatory guidelines and microbiological cut-off values.

## Data Availability

The raw data supporting the conclusions of this article will be made available by the authors on request.
